# Principles and Practice of Filling Teeth

**Published:** 1867-09

**Authors:** WM. O. Kulp

**Affiliations:** Muscatine, Iowa


### PRINCIPLES AND PRACTICE OF FILLING TEETH.

BY WM. O. KULP, D. D. S., MUSCATINE, IOWA.

MAN is not excluded from all nature's end—decay, both in mind and body. This is the tendency of all God's creatures on the earth; but man seems to be the only one that does so prematurely, and as all things were created for man, God has prepared remedies in some shape for all our ills, and given us science, by the use of which we can unfold these treasures and then, if they are timely and properly applied they *will* cure. It is not less so in the case of decayed teeth than in any other of man's diseases. I will only treat of the mechanical, or surgical means of arresting this disease, not stopping to discuss the cause of decay, or its *modus operandi* of destruction, or the possibility of arresting it by physiological means, as that is to be discussed as per programme, when we are through with the essays and their discussion.

In the process of saving teeth by filling them, are more shipwrecks than in any other branch of our profession; notwithstanding there has been more said and written on it. The question naturally arises, why is this the case. We must conclude that the fault must be in having so many in our profession who are wholly unfit, naturally and by education, to fill teeth; and they should not be allowed to continue to make efforts in that direction—a class who are honest men, who can make a very fair set of teeth, yet *cannot* possibly fill a tooth so as to save it. These ought to take up their specialty, or leave Dentistry to those whom nature has endowed with better qualifications.

Another class that fail constantly and more ignominiously than the former, are those who have not made Dentistry their study, but whom the painter and printer have made such, and they have nothing to expect but failure. If this class, and it is a large one, could be gotten rid of, there would be a grand gala day for the natural teeth. There would be fewer cheap sets of teeth worn. But these are not all who fail in saving teeth by filling: many who practice Dentistry, have not health and strength sufficient to do it well. It is a too important operation for such men to undertake; and men who have not the physical power to endure the hardships and confinement, should not be allowed to come into the profession. If a man is true to his profession, let it be Dentistry or any other, he will have to labor zealously and hard; and those who will not do this, or who are unable, should not choose a profession. Then there is a class who fail, half the time or more, and who have been in the profession so long, that we are wont to give them honor for it; yet the fact is, this is the class that makes failures respectable; and, in my opinion, the great reason why they are making so many failures is, because they are *behind the times.* They depend on their "many years" in the profession, rather than on real skill. Jealousy is a great controlling power in their organization; and for fear that they might be known to have learned something of some one younger in the profession than themselves, they will leave unlearned that which would be the secret of success in the very points wherein they now fail. Such bigotry is not becoming in a member of the Dental profession. It is the spirit that says, "*no improvement* in anything, the old way is the best, and the only one admissible." Any one possessing this element, should not be admitted into offices as a student; and if such are already in the profession, they ought to have help to get over that unfortunate obstacle, or be expelled from our ranks.

There are a great many failures made in the hands of much better men than any that I have yet enumerated; and it is because of neglect in some little point; and the object of my essay shall be to refer to points of error, more than to give the usual and oft-repeated process of filling teeth. More failures are made in dressing out cavities than in filling. How many will leave softened dentine in the cavity, intentionally, and from want of proper care! Often there is a strip of this dentine immediately beneath the enamel, and then again in the bottom of the cavities. How often is this left unclean! And then, how often are the margins of the cavities left rough and frail, so that it is impossible to make a filling permanently impervious, if at all, as the edges are apt to break off in the operation of filling. Or if not then, they will soon after, and thus leave a roughness around the cavity or filling, which causes a receptacle for foreign substances, and consequent destruction of all that has been done. In the cases first enumerated, I would urge that all the decayed dentine be thoroughly removed, so that the walls of the cavity are healthy bone, and so shaped that if a filling is properly put in, it will be philosophically impossible for it to come out; and the margin of the cavity should be cut down until a strong thick wall is obtained, and then it ought to be beveled or rounded, convex shaped and perfectly polished, and the gold be brought over the margin, and thoroughly condensed and polished off; the result is that the tooth is *restored*, in *all particulars*, and if kept clean and polished, the tooth is *permanently saved.* There is still another form of cavities in which a great many failures are made. Take, for instance, either a molar or bicuspid, in which there is decay in the centre of the crown, or both in the centre and oppositional surfaces. In either case, all fissures should be thoroughly explored, and cut out; and if there is an approximal, and central cavity, both should be cut into one cavity, and filled as one. The same rules of polishing the margins, and thoroughly cleansing cavity, should be observed. In all cases, a filling is not first class, that is not well polished, and a perfect fit made at margins of cavity and filling, and well polished. I tell you, gentlemen, generally there are not enough pains taken in the whole operation. Low prices have made many a poor Dentist, in more ways than one. Let *us* be honest men, and *do well* and *thoroughly all* that we undertake; and the public *will* pay us well for it. *Real merit* has its reward not less in Dentistry than in other pursuits of life. Let us not be fearful that we get too much gold into a cavity, or that we spend too much time on a filling or cavity. Let our motto be, do *well*, do *thoroughly*, and finish perfectly all that we undertake; and my word for it, we will get rich, and be honored by those whom we serve.

